# An Improved pH-Driven Method for Upcycling Polyphenols from Plants or Byproducts into Foods

**DOI:** 10.3390/foods13233945

**Published:** 2024-12-06

**Authors:** Xiping Gong, Minghe Wang, Peng Lu, Hualu Zhou

**Affiliations:** 1 Department of Food Science and Technology, College of Agricultural and Environmental Sciences, University of Georgia, Griffin, GA 30223, USA; xipinggong@uga.edu (X.G.); minghe.wang@uga.edu (M.W.); 2 Department of Agricultural Leadership, Education and Communication, College of Agricultural and Environmental Sciences, University of Georgia, Athens, GA 30602, USA; penglu@uga.edu

**Keywords:** alkali–acid treatment, upcycling, peanut skin, food nanoemulsions, byproducts

## Abstract

The incorporation of polyphenols into food systems provides various health benefits, yet their stability and bioactivity are often compromised by processing conditions. In this study, we advanced the pH-driven method for processing highly pH-sensitive polyphenols, such as quercetin, by optimizing operating conditions, including minimizing oxygen exposure and reducing operating times. As a result, an improved post-pH-driven (PPD) method was developed to encapsulate pH-sensitive quercetin into nanoemulsions with an encapsulation efficiency exceeding 95%, indicating that this method could be broadly applicable for incorporating various polyphenols. For example, it has been successfully applied to upcycle plant polyphenols from peanut skin into nanoemulsions, serving as a representative food model. The PPD method demonstrated superior performance compared to a conventional water-based method, achieving 1.8 times higher remaining percentage of total polyphenolic content. Additionally, the PPD-based nanoemulsions exhibited significantly enhanced antioxidant properties, with DPPH and ABTS radical scavenging activities increasing by 3.7 and 2.8 times, respectively, compared to the water-based method. These findings underscore the potential of the PPD method as a versatile and efficient approach for developing polyphenol-powered foods by upcycling plant byproducts and improving processing efficiency.

## 1. Introduction

The projected global population of approximately 10 billion by 2050 poses a significant challenge to achieving food sustainability and environmental preservation. At the same time, emerging technologies have heightened expectations for future foods, emphasizing health, nutrition, flavor, and cost-effectiveness [1]. Polyphenols, which are unique components found in plants like fruits, vegetables, tea, and coffee, represent a valuable resource for developing premium food products [2]. These compounds have been proposed as potential food additives that can improve the quality, shelf life, nutritional content, and safety of various food and beverage products [3,4]. Many of these foods operate as colloidal dispersions, consisting of proteins, carbohydrates, and lipids distributed within an aqueous matrix [5]. This composition naturally facilitates the encapsulation and delivery of polyphenols. Lipophilic polyphenols (LPs) can be encapsulated within the hydrophobic phase of oil bodies in foods, while hydrophilic polyphenols can be protected by proteins or polysaccharides [6]. For example, plant-based (PB) foods, such as PB milks made from nuts or beans, naturally contain oil bodies along with biomolecules like plant proteins and polysaccharides, making them excellent carriers for polyphenols [7,8,9].

The incorporation of polyphenols into food products has shown great potential for developing health-promoting foods and ingredients, offering benefits such as enhanced nutritional profiles, improved appearance, and extended shelf life [10]. For instance, oil bodies present in plant-based milks can protect LPs from chemical degradation or physical crystallization under harsh conditions [11]. Additionally, polyphenols, valued for their natural coloring properties, offer a flexible solution for enhancing the appearance of foods [12]. For instance, curcumin is commonly used as a yellow pigment and can be utilized to produce egg analogs with the desired coloration [13]. Beyond their esthetic contributions, plant polyphenols are highly valued for their antioxidant and antibacterial properties, which contribute to extending the shelf life of foods [14]. Their antioxidant properties help reduce oxidation and delay fat spoilage in meat products by neutralizing free radicals and preventing lipid peroxidation, thereby maintaining quality. Numerous studies have demonstrated the benefits of polyphenols, including their antioxidative, anti-obesity, antidiabetic, antiproliferative, and anti-inflammatory effects [7]. Epidemiological studies indicate that diets rich in polyphenols are associated with a reduced risk of chronic diseases, such as diabetes and neurodegenerative disorders [15].

Many plant byproducts, such as peanut skins, are notably rich in bioactive compounds like polyphenols, which offer antioxidant and health-promoting benefits [16,17]. Upcycling polyphenols from these byproducts provides a sustainable solution to valorize waste materials while enhancing the nutritional and functional properties of food products [18,19,20]. This approach not only reduces waste but also enables the creation of nutrient-enriched products that align with current health and environmental trends. However, achieving a practical solution for incorporating polyphenols from plants and byproducts into high-quality food production remains challenging. A major hurdle is the sensitivity of many polyphenols to processing factors such as light, oxygen, temperature, and pH, which can lead to their degradation and reduced efficacy during production [21]. For instance, high-temperature processing can cause undesirable darkening in polyphenol-containing foods, and the addition of lipophilic polyphenols can lead to low water solubility, crystallization, and reduced bioavailability. These challenges arise from the reactive structural groups in polyphenols (such as phenolic hydroxyl, C=C double bonds, and ketones), which are sensitive to pH, prone to chemical degradation, and capable of non-covalent interactions with biomolecules [21].

An efficient production strategy is essential for upcycling polyphenols from plants or byproducts to enhance the functional qualities of foods [22]. Currently, polyphenol extraction and encapsulation are separate processes, leaving a knowledge gap in reducing energy and resource consumption. Extracting and encapsulating polyphenols is challenging due to issues like crystallization, chemical degradation, and their entrapment in plant tissues. Conventional organic solvent-based extraction methods are inefficient, time-consuming, and environmentally harmful, consuming excessive samples, energy, and solvents, which underscores the need for more eco-friendly and sustainable green techniques [23]. Encapsulation of polyphenols, particularly LPs, also presents challenges due to their low water solubility [24]. Two common methods—heat-driven and organic solvent-driven—are typically used. In the heat-driven method, LPs are either dispersed in the oil phase prior to homogenization or dissolved into a delivery system, such as nanoemulsions. Heating LPs (e.g., curcumin at 100 °C) is commonly employed to aid dissolution; however, this process can cause thermal degradation or clogging of homogenizer channels if dissolution is incomplete [25]. Additionally, it can take considerable time to break intermolecular interactions and fully solubilize LPs into the oil phase, resulting in high energy costs. Alternatively, LPs can be dissolved in organic solvents and introduced into an anti-solvent phase, such as water, to facilitate their transfer into the hydrophobic phase of the delivery system [26]. However, the use of organic solvents is less desirable because of their environmental impact and the high cost associated with their removal.

Recently, the pH-driven method for polyphenol extraction and encapsulation in food systems has gained attention due to its simplicity, speed, and lack of need for sophisticated equipment. The pH-driven method has been reviewed for encapsulating polyphenols into nanoemulsions and other delivery systems [24,27,28,29]. The principle relies on the pH-dependent solubility of polyphenols. Under acidic and neutral conditions, LPs are uncharged, hydrophobic, and poorly soluble in water. However, raising the pH leads to the deprotonation of phenolic hydroxyl groups, increasing hydrophilicity and water solubility [30]. Various pH-driven approaches have been used to introduce LPs into food-grade delivery systems [24,27]. An early study demonstrated the dissolution of curcumin and sodium caseinate in a NaOH solution (pH 12), followed by neutralization with HCl, resulting in the formation of curcumin-loaded casein micelles [31]. This method enables simultaneous particle formation and LP incorporation and has gained widespread adoption. Delivery systems such as micelles [32,33], liposomes [34], nanogels [35,36], and biopolymer nanoparticles [28,37,38] have been fabricated using this approach. Initially developed for curcumin [31], the method has also been applied to other polyphenols and drug molecules, including quercetin [39], resveratrol [40], rutin [41], and eugenol [42]. An alternative pH-driven approach has been developed, where polyphenols are loaded after the delivery system is formed. This method has been applied to micelles [43], liposomes [33,44], and oil-in-water emulsions or nanoemulsions [11,45]. This method is becoming increasingly popular for incorporating a wide range of polyphenols due to its versatility, such as curcumin [46,47], resveratrol [11], and quercetin [30], into colloidal delivery systems. Studies have demonstrated that the pH-driven approach can effectively incorporate polyphenols into preformulated systems, particularly in bovine milk and plant-based milk [46,47,48,49,50]. A recent study has provided valuable structural insights into the pH-driven method, demonstrating that it can significantly alter the structure of crystalline LPs, thereby enhancing their solubilization into delivery systems [51].

Unfortunately, we still face several challenges in integrating the pH-driven method for practical applications in the development of polyphenol-powered foods. First, current pH-driven approaches are not universally applicable to many polyphenols, particularly those that are highly pH-sensitive [30]. This sensitivity arises from the phenolic OH groups, which are prone to degradation in alkaline solutions. While conventional pH-driven methods can achieve relatively high encapsulation efficiencies for certain compounds, such as curcumin and resveratrol, through rapid encapsulation [11], they perform poorly with polyphenols that possess highly pH-sensitive structures. For instance, quercetin, due to the presence of an unstable 3-hydroxyflavone backbone, exhibits a lower encapsulation efficiency (60–70%) due to its structural vulnerability [11,30]. This is primarily due to improper treatment conditions often employed in conventional pH-driven approaches, which fail to adequately protect sensitive polyphenols. Second, research on integrating both extraction and encapsulation is still in its early stages, with most studies focusing on encapsulation applications [29]. For example, current encapsulation research often begins with purified bioactive compounds, overlooking the potential to streamline the production pipeline by extracting and encapsulating bioactive compounds directly from raw plant materials or byproducts. This gap highlights the need to develop methodologies that combine these two steps, enabling simultaneous extraction and encapsulation to improve efficiency, reduce processing time, and lower resource consumption. Such integrated approaches could also minimize waste, maximize the utilization of raw materials, and enhance the sustainability of functional foods.

In this work, we propose an improved pH-driven method to address these challenges. This method aims to incorporate polyphenols from plants or byproducts into food systems while achieving high encapsulation or remaining efficiency, even for highly pH-sensitive polyphenols. This approach builds upon the conventional pH-driven method [48], with key modifications. Specifically, the improvements to minimize polyphenol degradation include (a) using a confined container to limit oxygen and light exposure and (b) significantly reducing the time polyphenols remain under highly alkaline conditions by adjusting pH and optimizing operating conditions, such as rapid dissolution. Additionally, this method will integrate both extraction and encapsulation steps to achieve its practical potential and feasibility. We believe this improved pH-driven method can be effectively applied to upcycle polyphenols from plants or byproducts, contributing to the development of polyphenol-powered foods.

## 2. Materials and Methods

### 2.1. Materials

The chemicals purchased from TCI America (Tokyo, Japan) include quercetin (purity > 96.0%) and 6-hydroxy-2,5,7,8-tetramethylchroman-2-carboxylic acid (Trolox, purity > 98.0%). The chemicals purchased from Sigma-Aldrich (St. Louis, MO, USA) include citric acid (CAS number: 77-92-9, ≥99.5%), Folin–Ciocalteu’s phenol reagent, Tween 20, and potassium persulfate (K_2_S_2_O_8_). The chemicals purchased from Thermo Fisher Scientific (Hampton, NH, USA) include 1.0 N hydrochloric acid solution (HCl, CAS number: 7647-01-0, certified ACS reagent grade), sodium hydroxide powder (NaOH, purity > 98.0%, certified ACS reagent grade), gallic acid, 2,2-diphenyl-1-picrylhydrazyl (DPPH), 2,2′-azino-bis (3-ethylbenzothiazoline-6-sulfonic acid) (ABTS), sodium carbonate, acetone, and acetic acid. The corn oil (Mazola, ACH Food Company, Memphis, TN, USA) was purchased from a local grocery store. Peanuts (Georgia-06G) were obtained from the Georgia Tifton campus (Tifton, GA, USA). The ethanol used was analytical grade, and the acetic ethanol (1 wt%) used was prepared using an ethanol solution containing 1% acetic acid. Double-distilled water was used throughout this study.

### 2.2. Dissolution of Quercetin Under Different Operating Conditions

To examine the dissolution of quercetin under varying pH levels, quercetin (30 mg) was dissolved in 5 mL of NaOH solutions with pH values ranging from 8 to >14 (6 N). The effects of different initial quercetin concentrations (0.1, 2, and 15 mg/mL), oxygen exposure levels (controlled by using containers with varying air exposure: an open-top beaker, a 15 mL closed tube, and a 2 mL closed tube), and operating times (0.5, 1, 2, 3, 4, 6, 8, 10, and 30 min) were also investigated.

### 2.3. Making Peanut Skin Powder

Five pounds of peanuts were lightly baked in a pilot-scale oven (Lincoln Impinger, Lincoln Foodservice Products, Fort Wayne, IN, USA) at 325 °F for 6 min to loosen the skins from the kernels. After baking, the peanuts were passed through a blancher (Ashton Food Machinery Co., Newark, NJ, USA) to separate the skins from the kernels. The collected peanut skins were then ground into a fine powder using a nut grinder (Cuisinart, Stamford, CT, USA). The resulting powder was sieved through a sieve to ensure a uniform particle size. This procedure was repeated for three independent trials, each using five pounds of peanuts. The peanut skin powder from each trial was stored in airtight containers at refrigerated temperatures to prevent oxidation and moisture absorption until further analysis.

### 2.4. Preparation of Nanoemulsions and the Incorporation of Quercetin or Peanut Skin

Initially, 5.00 g of emulsifier (Tween 20) was added to a 1000 mL glass beaker, followed by the addition of distilled water to bring the total sample weight to 450.00 g. After the emulsifier was fully dissolved, 50.00 g of corn oil was introduced, creating an initial sample with 10 wt% oil and 1 wt% emulsifier. The sample was mixed for 5 min using a laboratory homogenizer (Omni International, Kennesaw, GA, USA) to form coarse emulsions. The coarse emulsions were processed through a microfluidizer (LM20, Microfluidics, Westwood, MA, USA) at 12,000 psi for three cycles to create stable nanoemulsions.

To incorporate quercetin into the nanoemulsions, 2.0 mL of NaOH solution (pH 12) was used to dissolve 30 mg of quercetin in a 2 mL closed tube during 2 min of vortexing. The dissolved quercetin solution was then added directly to 50 mL Tween 20-stabilized nanoemulsions, followed by rapid agitation to ensure thorough mixing. At the same time, an acidification process was rapidly applied to neutralize the mixture until the pH reached 7 by using 0.1 N HCl. The final quercetin concentration was 6 mg/g oil. In addition, we used a conventional pH-driven method to incorporate the same amount of quercetin into nanoemulsions. However, unlike previous studies where quercetin was dissolved in an open beaker for ~10 min, we utilized a 2 mL closed tube to minimize oxygen exposure during dissolution [11,45].

To incorporate peanut skin into the nanoemulsions, a similar protocol was followed. Fresh peanut skin (0.5 g) was weighed and put into a 15 mL test tube. Then, 15.0 mL of 0.1 N NaOH solution or water was added to the test tube, and the sample was centrifuged at 5000 rpm for 10 min. The sample was labeled as a PPD-based or water-based extraction. Moreover, 5.0 mL of supernatant of either PPD-based or water-based samples was added into 20 mL of nanoemulsions. At the same time, an acidification process was rapidly applied to neutralize the mixture until the pH reached 7 by using 0.1 N HCl. The volume of the sample was finally adjusted to 40.0 mL by adding water, and the sample was labeled as either PPD-based or water-based.

### 2.5. Determination of Quercetin Concentration

The concentration of quercetin in each sample was measured using a UV–Vis spectrophotometer (Genesys 150, Thermo Fisher Scientific, Waltham, MA, USA) at 370 nm by following prior studies [30,52]. Prior to measurement, each sample was diluted with acidic ethanol (1% acetic acid) to achieve a suitable concentration. The diluted solution was then centrifuged at 4000 rpm for 2 min. After centrifugation, the supernatant was transferred to a quartz cuvette and measured at 370 nm. A standard curve for quercetin was established using a series of solutions with known polyphenol concentrations (y = 0.0725x + 0.0016, *R*^2^ = 0.9999), where x is quercetin concentration (in μg/mL) and y is the corresponding absorbance value. The encapsulation efficiency of quercetin was calculated by following a prior study [11].

### 2.6. Appearance, Particle Dimensions, and Surface Charge

A colorimeter (ColorFlex EZ 45/0-LAV, Hunter Associates Laboratory Inc., Reston, VA, USA) was used to measure the color of the peanut skin-loaded nanoemulsions. The L*, a*, and b* values were measured by pouring 10 mL of the sample into a transparent Petri dish. The black and white standardized plates were used as the blanks with the standardized light source (D65) and detection angle (10 degrees). The L* value of the sample was measured to assess their lightness or opacity, the a* value to indicate their redness/greenness (+/−), and the b* value to represent their yellowness/blueness (+/−). Photos were taken using an iPhone 13 camera with a black background and white light. Particle size and zeta potential were analyzed using dynamic light scattering (DLS) with a Zetasizer Pro (Malvern Panalytical, Malvern, UK). Measurements were taken without dilution to preserve the original concentration and conditions.

### 2.7. Total Phenolic Content (TPC)

The TPC of fresh peanut skin and various liquid samples was measured using a previously reported method with slight modifications [53]. To determine the TPC, fresh peanut skin powder (5 g) and liquid samples (1 mL, including PPD-based extraction, water-based extraction, PPD-based remaining, and water-based remaining) were placed into a centrifuge tube. An extraction solution (5 mL) consisting of acetone/water/acetic acid (70:29.5:0.5, *v*/*v*/*v*) was added, and the mixture was shaken on an orbital shaker (Thermo Fisher Scientific, Waltham, MA, USA) for 3 h, followed by 12 h of shaking in the dark. The mixture was then centrifuged at 5000 rpm for 10 min. This extraction process was repeated twice, and the final volume of the extract was collected and recorded. For TPC analysis, 50 μL of the collected sample was mixed with 250 μL of Folin–Ciocalteu solution and stored in the dark for 5 min. Next, 750 μL of 7% sodium carbonate solution was added, and the mixture was incubated in the dark for another 5 min. Finally, 950 μL of distilled water was added, the solution was vortexed, and it was incubated in the dark for 1 h. Absorbance was measured at 765 nm using a spectrophotometer (Genesys 150, Thermo Fisher Scientific, Waltham, MA, USA). The gallic acid was used to make the calibration curve, y = 0.0009 x + 0.0015 (*R*^2^ = 0.9994), where x is the gallic acid concentration (in μg/mL), and y is the corresponding absorbance value. The TPC result was reported as micrograms of gallic acid equivalents per gram solid sample (μg GAE/g) or per milliliter liquid sample (μg GAE/mL). The extraction and remaining percentages using either the PPD-based or water-based method are defined as follows, respectively:

(1) $$\mathrm{Extraction}\;(\%)=\frac{{TPC}_{\left(PPD-based\;extration\;or\;Water-based\;extration\right)}}{{TPC}_{\left(fresh\;peanut\;skin\right)}}$$

(2) $$\mathrm{Remaining}\;(\%)=\frac{{TPC}_{\left(PPD-based\;remaining\;or\;Water-based\;remaining\right)}}{{TPC}_{\left(fresh\;peanut\;skin\right)}}$$

where the TPC value of fresh peanut skin powder was 95.4 mg GAE/g. The TPC of fresh peanut skin was calculated by the product of their total phenolic concentration and weight, while the TPC of liquid samples was calculated by the product of total phenolic concentration and their corresponding volume. The extraction efficiency quantifies the percentage of polyphenols successfully extracted, while the remaining efficiency represents the percentage of polyphenols that are both extracted and encapsulated into the final food product. Consequently, encapsulation efficiency is defined as the ratio of remaining efficiency to extraction efficiency.

### 2.8. DPPH-Free Radical Scavenging Activity Assay

The DPPH values in fresh peanut skin and different liquid samples were determined by following a previously reported method [53]. To determine the DPPH value, fresh peanut skin powder (5 g) and liquid samples (1 mL, including PPD-based extraction, water-based extraction, PPD-based remaining, and water-based remaining) were placed in centrifuge tubes, and 5 mL of extraction solution (acetone/water/acetic acid, 70:29.5:0.5, *v*/*v*/*v*) was added. The mixture was shaken on an orbital shaker for 3 h, followed by 12 h of shaking in the dark. Afterward, the mixture was centrifuged at 5000 rpm for 10 min. This extraction process was repeated twice, and the final extract volume was collected and recorded. Diluted extract samples (0.4 mL) were then mixed with freshly prepared DPPH solution (7.6 mL) and allowed to react at room temperature for 30 min. Absorbance was measured at 517 nm using a spectrophotometer (Genesys 150, Thermo Fisher Scientific, Waltham, MA, USA). The Trolox was used to make the calibration curve, y = 0.001 x − 0.0129 (*R*^2^ = 0.9992), where x is the Trolox concentration (in μM /TE) and y is the corresponding absorbance value. The DPPH scavenging activity of the sample was reported as micromoles of Trolox equivalent per gram solid sample (μmol TE/g) or per milliliter liquid sample (μmol TE/mL). The extraction and remaining percentages using either the PPD-based or water-based method are defined as follows, respectively:

(3) $$\mathrm{Extraction}\;(\%)=\frac{{DPPH\;value}_{\left(PPD-based\;extration\;or\;Water-based\;extration\right)}}{{DPPH\;value}_{\left(fresh\;peanut\;skin\right)}}$$

(4) $$\mathrm{Remaining}\;(\%)=\frac{{DPPH\;value}_{\left(PPD-based\;remaining\;or\;Water-based\;remaining\right)}}{{DPPH\;value}_{\left(fresh\;peanut\;skin\right)}}$$

where the DPPH value of fresh peanut skin was 568.5 μmol TE/g. The DPPH value of fresh peanut skin was calculated by the product of their total phenolic concentration and weight, while the DPPH value of liquid samples was calculated by the product of their total phenolic concentration and their corresponding volume.

### 2.9. ABTS Radical Scavenging Assay

The ABTS value of fresh peanut skin and various liquid samples was measured following a previously reported method [54]. To determine the ABTS value, fresh peanut skin powder (5 g) and liquid samples (1 mL, including PPD-based extraction, water-based extraction, PPD-based remaining, and water-based remaining) were placed into centrifuge tubes, and 5 mL of extraction solution (acetone/water/acetic acid, 70:29.5:0.5, *v*/*v*/*v*) was added. The mixture was shaken on an orbital shaker for 3 h, followed by 12 h of shaking in the dark. The mixture was then centrifuged at 5000 rpm for 10 min. This process was repeated twice, and the final extract volume was recorded. The processed sample and ABTS solution were diluted with ethanol. Diluted extract samples (60 μL) were mixed with the freshly prepared ABTS solution (3 mL) and incubated for 6 min at 30 °C. Absorbance was measured at 734 nm using a spectrophotometer (Genesys 150, Thermo Fisher Scientific, Waltham, MA, USA). The Trolox was used to make the calibration curve, y = −0.0005 x + 0.5873 (*R*^2^ = 0.9963), where x is the Trolox concentration (in μM /TE) and y is the corresponding absorbance. The ABTS radical scavenging activity of the sample was reported as micromoles of Trolox equivalent per gram solid sample (μmol TE/g) or per milliliter liquid sample (μmol TE/mL). The extraction and remaining percentages using either the PPD-based or water-based method are defined as follows, respectively:

(5) $$\mathrm{Extraction}\;(\%)=\frac{{ABTS\;value}_{\left(PPD-based\;extration\;or\;Water-based\;extration\right)}}{{ABTS\;value}_{\left(fresh\;peanut\;skin\right)}}$$

(6) $$\mathrm{Remaining}\;(\%)=\frac{{ABTS\;value}_{\left(PPD-based\;remaining\;or\;Water-based\;remaining\right)}}{{ABTS\;value}_{\left(fresh\;peanut\;skin\right)}}$$

where the ABTS value of fresh peanut skin was 819.3 μmol TE/g. The ABTS value for solid samples was evaluated by multiplying the total phenolic concentration by the weight of the peanut skin, while for liquid samples, it was determined by multiplying the total phenolic concentration by the sample’s corresponding volume.

### 2.10. Statistical Analysis

At least three measurements were performed for all experiments, and the mean and standard error were then calculated. One-way ANOVA, followed by a post hoc Tukey HSD test (*p* < 0.05), was used to determine statistical significance [55].

## 3. Results and Discussion

### 3.1. Impact of Operating Conditions on the Stability of pH-Sensitive Quercetin

While the pH-driven method offers advantages in improving the solubility and encapsulation efficiency of phenolic compounds, it has a notable limitation: the instability of pH-sensitive compounds at extreme pH levels (e.g., pH 12). Under such highly alkaline conditions, these compounds can degrade, resulting in a loss of bioactivity and reduced functional properties. In this study, quercetin was selected as a representative compound to address this limitation for several reasons. First, previous research has shown that the stability of quercetin during processing is significantly affected by factors such as pH and oxygen exposure, and it has been suggested that the conventional pH-driven method is not suitable for highly pH-sensitive polyphenols like quercetin [30]. Second, quercetin is widely considered a model polyphenol due to its structure. As a flavonoid, it contains five hydroxyl (-OH) groups on its phenolic rings, which contribute to its antioxidant activity—a feature common to many polyphenols. This makes quercetin an ideal compound for studying polyphenolic behavior. Third, quercetin is abundant in a variety of fruits, vegetables, and grains, such as apples, onions, berries, and tea, and its bioactivity makes it a key polyphenol of interest in food science. Understanding how the operating conditions affect the stability of quercetin is therefore crucial for applying this method to upcycle plant byproducts into food systems.

**Figure 1:**
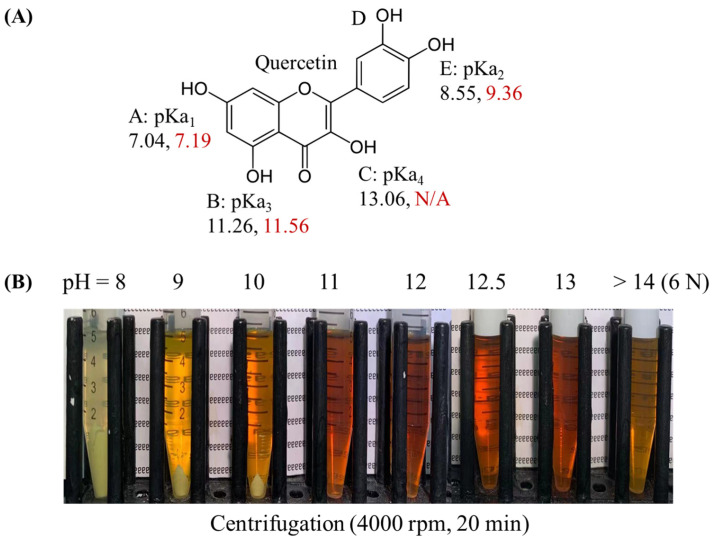
Impact of pH on quercetin solubility under alkaline conditions. (**A**) The pKa values of the quercetin molecule, obtained from a previous study, including computational predictions (in black) and experimental measurements via capillary electrophoresis (in red), N/A means not available [56]. (**B**) Photographs of quercetin (30 mg) solubilized in 5 mL of alkaline solutions at different pH levels, following centrifugation at 4000 rpm for 20 min.

In this study, we investigated the effects of controlled operating conditions, specifically pH levels and oxygen exposure, on the degradation and retention of quercetin. The pH-driven method operates by adjusting the pH to alter the solubility of phenolic compounds, allowing them to take on negatively charged forms. Previous studies have reported pKa values of quercetin, which include multiple pKa values derived from computational predictions and experimental measurements (Figure 1A) [56]. We dissolved 25 mg of quercetin powder into 5 mL of alkaline solutions at different pH levels (Figure 1B), observing distinct color changes that corresponded to progressive deprotonation and possible degradation of quercetin as pH increased. At lower pH values (e.g., pH < 11), quercetin dissolution was incomplete, as evidenced by undissolved powder at pH 8–10. Higher pH values, ranging from pH 11 to 13, effectively dissolved the quercetin powder. However, in extremely alkaline conditions (6 N), a noticeable color change indicated significant structural alteration due to deprotonation of the hydroxyl group on the C-ring of quercetin, leading to rapid degradation. These observations underscore the importance of selecting an appropriate pH level when applying the pH-driven method to real-world applications.

In addition to pH, oxygen exposure is a critical factor in the degradation of quercetin. Like many polyphenols, quercetin is highly susceptible to oxidation when exposed to oxygen, which leads to the formation of quinones and other degradation products, thereby reducing its antioxidant capacity [57]. Minimizing oxygen exposure during processing is, therefore, essential to preserving the stability and bioactivity of quercetin, particularly when combined with pH adjustments. While numerous studies have highlighted the importance of controlling oxygen exposure to mitigate quercetin degradation, a straightforward and practical processing condition for real-world applications of the pH-driven method has yet to be established. Specifically, there remains a need to determine whether a >90% encapsulation efficiency can be achieved under controlled yet feasible operating conditions.

**Figure 2:**
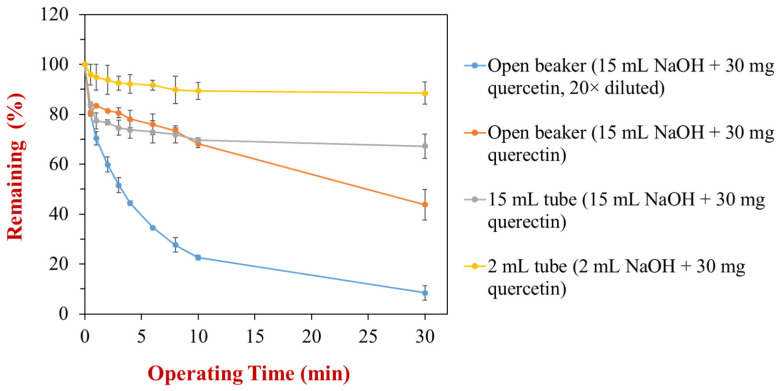
Impact of varying oxygen exposure levels and operating time on the chemical stability of quercetin at pH 12. Different concentrations of NaOH (e.g., 0.1 N or 1.0 N) were used to adjust the final volume and achieve the target pH value. ‘20× diluted’ indicates that the mixed solution was diluted 20-fold in a pH 12 NaOH solution.

We investigated three critical factors—initial quercetin concentration, oxygen exposure level, and operating time—that affect quercetin stability in a pH 12 alkaline solution (Figure 2). The results show that the chemical stability of quercetin is closely linked to these factors. At a low initial concentration (0.1 mg/mL), quercetin undergoes rapid exponential degradation due to constant air exposure. Increasing the initial quercetin concentration to 2 mg/mL slows the degradation rate, but quercetin still degrades quickly. This explains why the conventional pH-driven method has low encapsulation efficiency for quercetin, as it fails to account for the impact of air exposure on degradation. However, in closed containers, degradation follows a linear pattern due to reduced oxygen exposure. For example, using confined containers like 15 mL or 2 mL tubes significantly mitigates degradation, with the 2 mL tube showing the most notable improvement. In a confined 2 mL tube, more than 90% of the quercetin was retained after 30 min, suggesting that minimizing oxygen exposure by using confined containers effectively reduces chemical degradation. Overall, two practical suggestions can improve the processing efficiency of the pH-driven method: minimizing oxygen exposure and increasing the initial concentration of quercetin. This highlights the critical role of the polyphenol-to-oxygen concentration ratio in reducing degradation.

### 3.2. An Improved pH-Driven Approach

**Figure 3:**
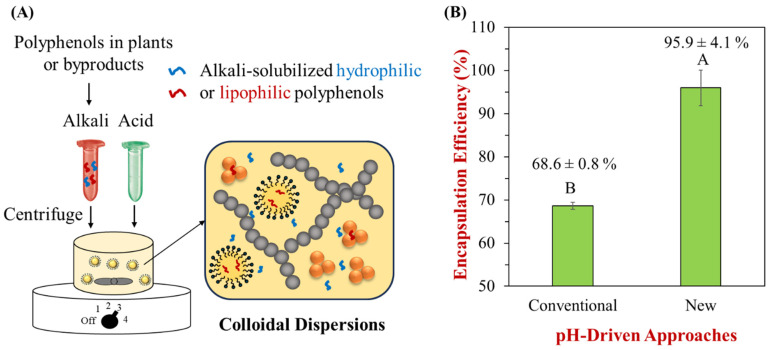
Improved encapsulation efficiency of polyphenols by minimizing oxygen exposure. (**A**) Schematic representation of the improved PPD approach for incorporating polyphenols from plants or byproducts into colloidal dispersions. (**B**) Comparison of the encapsulation efficiency of quercetin in nanoemulsions using the conventional pH-driven approach and the new PPD approach. The conventional pH-driven approach was based on the procedure described in previous studies [11,45]. Uppercase letters (A and B) indicate significant differences between samples (*p* < 0.05).

Based on the preliminary investigations, an improved pH-driven method, termed the post-pH-driven (PPD) method, was proposed to incorporate polyphenols from plants or byproducts into food systems. This method advances the conventional pH-driven approach for developing polyphenol-powered foods. The PPD method is expected to significantly enhance processing sustainability and efficiency by streamlining processing steps and controlling conditions. For example, sustainability is improved through a two-in-one process that combines extraction and encapsulation in a single step, allowing for more efficient incorporation of polyphenols from plants or byproducts into foods (Figure 3A). Additionally, processing efficiency is increased by minimizing oxygen and light exposure and reducing the time polyphenols spend in an alkaline environment during the production of polyphenol-enriched foods.

### 3.3. Improved Encapsulation Efficiency of Very pH-Sensitive Quercetin in Nanoemulsions

Our preliminary results indicate that the proposed PPD method significantly improves encapsulation efficiency in the development of quercetin-encapsulated nanoemulsions (Figure 3B). The conventional pH-driven method has been widely used to encapsulate polyphenols or their combinations into food systems, such as nanoemulsions and dairy or plant-based milks. For example, a previous study encapsulated a mixture of curcumin, resveratrol, and quercetin into nanoemulsions, with respective encapsulation efficiencies (EE%) compared. Notably, the EE% varied: resveratrol (88%) ≈ curcumin (82%) > quercetin (61%), with a lower efficiency of quercetin attributed to its vulnerability to degradation in highly alkaline solutions (pH 12), largely due to inadequate consideration of oxygen exposure [52]. These findings underscored two key issues: the viability of encapsulating multiple polyphenols and the limitations of the conventional pH-driven approach for chemically sensitive compounds. Encouragingly, the proposed PPD method minimizes oxygen exposure and operates swiftly (<1 min), achieving an exceptional encapsulation efficiency (>95%) (Figure 3B). These results demonstrate a substantial improvement over the conventional pH-driven method.

The PPD approach stands out for its simplicity, as it does not require complex equipment or high-energy input, making it highly scalable for industrial production. Another key advantage is that raw plant materials or byproducts can be directly utilized, reducing the need for additional processing steps to extract polyphenols. This makes the PPD method promising for a two-in-one process, seamlessly integrating extraction and encapsulation into finished food products (Figure 3A). For example, the PPD approach offers an efficient route to incorporate polyphenols from peanut skin into food emulsions or milks. By combining extraction and encapsulation, the PPD method establishes itself as a more sustainable option, maximizing the utilization of plant materials while conserving energy. This improvement enhances the potential for developing polyphenol-powered novel foods. In contrast, existing studies often rely on isolated or purified polyphenol powders, typically extracted through organic solvent-based methods that are environmentally unfriendly and unsustainable. Directly using raw plant materials, therefore, provides a more sustainable and cost-effective solution compared to processed ingredients.

### 3.4. Upcycling Polyphenols from Peanut Skin into Nanoemulsions as a Food Model

The PPD method was applied to assess whether polyphenols derived from peanut skins could be effectively upcycled into food systems, with nanoemulsions serving as a representative food model. The simplicity of the PPD method was demonstrated through its straightforward processing steps. First, pH adjustment facilitated the extraction and solubilization of polyphenols directly into the aqueous phase. Furthermore, the ease of adjusting the pH back to neutral after encapsulation ensured the final product remained stable and compatible with various food matrices. The method required minimal processing equipment and avoided complex purification steps, highlighting its practicality for industrial applications. Overall, the PPD method provides a simple, scalable, and resource-efficient solution for incorporating peanut skin-derived polyphenols into nanoemulsified food systems.

**Figure 4:**
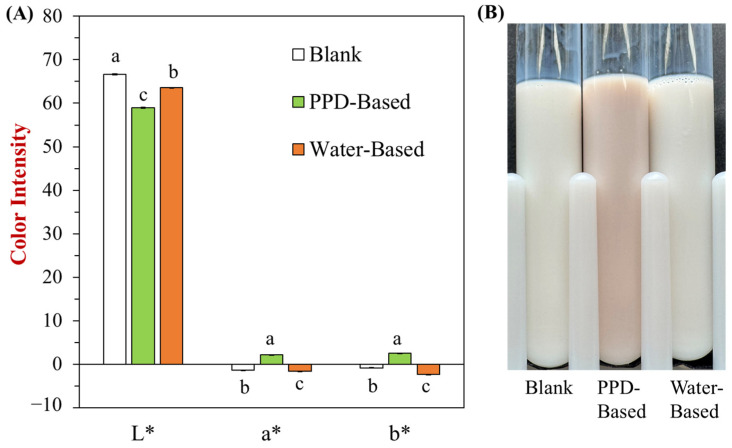
The appearance of peanut skin-powered nanoemulsions. (**A**) The color coordinates (L*, a*, and b*) of three different samples: Blank (nanoemulsions), PPD-Based (peanut skin-incorporated nanoemulsions via the new PPD method), and Water-Based (peanut skin-incorporated nanoemulsions via the water-based extraction method). (**B**) The photos of three different samples. The total color difference (ΔE) values between “Blank” and “PPD-based” or “Water-based” samples were 9.1 ± 0.2 and 3.4 ± 0.1, respectively, calculated as the square root of the sum of the squared differences in their L, a, and b values. Lowercase letters (a, b, and c) indicate significant differences between samples (*p* < 0.05).

To further investigate the impact of incorporating peanut skin on the physicochemical properties of nanoemulsions, we first measured the color change and appearance of peanut skin-incorporated nanoemulsions, comparing two different treatments: one using the PPD method and the other using water to extract and solubilize polyphenols into the nanoemulsions. The appearance of the peanut skin-incorporated nanoemulsions was visually appealing, with the nanoemulsions exhibiting a stable white hue and no visible phase separation or sedimentation over time. After incorporating polyphenols from peanut skins, the PPD-based sample showed a visible color change from white to pink, attributed to the natural pigments in the peanut skin-derived polyphenols. For instance, the blank sample had the highest L* value (whiteness), the PPD-based sample had the lowest L* value, and the water-based sample had a median L* value (Figure 4A). Additionally, the calculated total color difference (ΔE) values further indicate that the PPD-based sample differs from the water-based sample. The distinct difference in appearance between the PPD-based and water-based samples suggests that the PPD method incorporates more polyphenols from peanut skins into the nanoemulsions (Figure 4B). This highlights the effectiveness of the PPD approach in upcycling peanut skin polyphenols into food systems.

**Figure 5:**
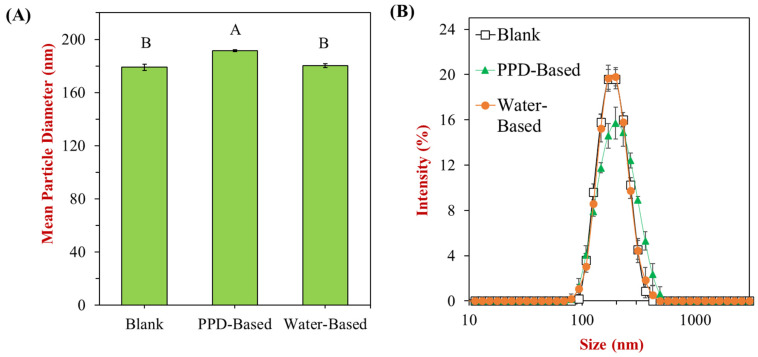
Impact of different treatments on the particle size of nanoemulsions. (**A**) Mean particle diameters and (**B**) size distributions of the three different samples. The PDI values are 0.059, 0.113, and 0.057, respectively. Uppercase letters (A and B) indicate significant differences between samples (*p* < 0.05).

We further investigated the impact of different treatments on particle size distribution to assess the effect of polyphenol incorporation on emulsion stability. DLS analysis revealed an average particle size of approximately 181 nm for the blank nanoemulsions. Incorporating peanut skin polyphenols using the water-based treatment had little effect on overall particle size, with a distribution similar to that of the blank nanoemulsions. However, using the PPD method resulted in polyphenol-incorporated nanoemulsions with a slightly larger mean particle diameter (~192 nm) compared to both the blank and water-based treatments. The PPD-based sample also exhibited a broader particle size distribution (Figure 5B), which may be due to the presence of larger particles, such as soluble peanut skin dietary fibers that dissolve in the alkaline solution and are incorporated into the nanoemulsions, potentially increasing particle size [58,59]. Despite this, all treatments produced monodisperse systems with low polydispersity index (PDI) values, indicating uniform droplet size and the absence of significant aggregation or coalescence. These findings suggest that the PPD method successfully integrates peanut skin polyphenols into nanoemulsions without significantly compromising particle size distribution, maintaining stability in food systems.

**Figure 6:**
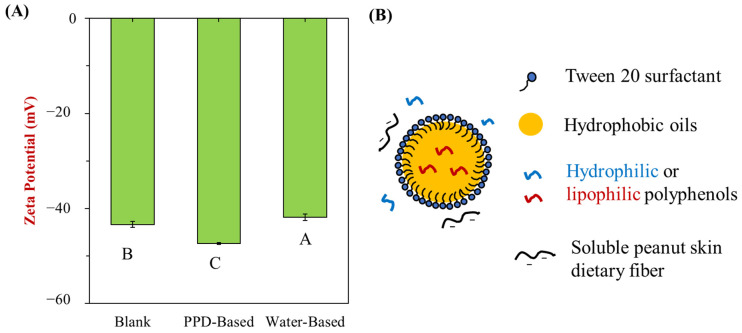
Impact of different treatments on the surface charges of nanoemulsions. (**A**) Zeta potentials of three different samples. (**B**) Proposed structures of peanut skin-incorporated nanoemulsions. Uppercase letters (A, B, and C) indicate significant differences between samples (*p* < 0.05).

Zeta potential measurements were performed to assess the surface charge of the nanoemulsions, providing insight into their stability. As shown in Figure 6A, the zeta potential values for the blank, PPD-based, and water-based nanoemulsions were all negative. The blank nanoemulsion exhibited a zeta potential of approximately −43 mV, indicating strong electrostatic repulsion between droplets due to the presence of Tween 20 surfactant. The PPD-based nanoemulsions, which incorporated peanut skin polyphenols, had a slightly higher zeta potential of around −47 mV, still within the range that ensures good stability. This suggests that the polyphenols did not significantly alter the surface charge, allowing for stable droplet dispersion. In contrast, the water-based nanoemulsion showed a slightly lower zeta potential of −42 mV, which was closer to that of blank nanoemulsions. Overall, the consistently negative zeta potential values across all treatments indicate sufficient electrostatic repulsion to maintain droplet separation, suggesting that the PPD method effectively incorporates polyphenols without compromising the colloidal stability of the nanoemulsions.

### 3.5. Improved Total Phenolic Content of Peanut Skin-Incorporated Nanoemulsions

**Figure 7:**
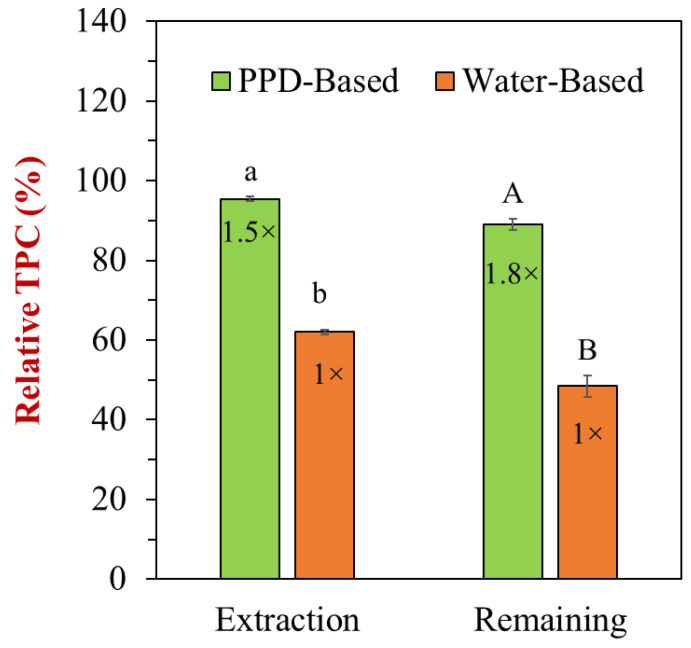
Impact of different treatments on the relative total phenolic content of peanut skin-incorporated nanoemulsions. Letters (a and b, or A and B) indicate significant differences between samples (*p* < 0.05). The ratio (e.g., “1.5×” or “1.8×”) was calculated relative to the water-based sample.

The relative TPC of peanut skin-incorporated nanoemulsions was significantly influenced by the treatment methods. The PPD-based nanoemulsions exhibited a notably higher TPC compared to the water-based method in both extraction efficiency and remaining phenolic content after processing. Specifically, the PPD-based nanoemulsions achieved approximately 1.5 times higher relative TPC than the water-based system, with the “PPD-Based” bar reaching nearly 100%, while the “Water-Based” bar remained at around 60% (Figure 7). This suggests that the PPD method was more effective in extracting polyphenols from peanut skins, likely due to its ability to alter the solubility of hydrophobic phenolic compounds under controlled alkaline conditions (pH 12). In terms of remaining phenolic content after processing, the PPD-based method retained approximately 1.8 times more TPC than the water-based method. This indicates that the PPD method not only improves the initial extraction but also enhances phenolic retention in nanoemulsions. Overall, these results demonstrate the superiority of the PPD method over the water-based method for both extracting and retaining phenolic compounds from peanut skin into nanoemulsions, making it a promising approach for increasing phenolic compounds in food systems.

### 3.6. Improved Antioxidant Properties of Peanut Skin-Incorporated Nanoemulsions

**Figure 8:**
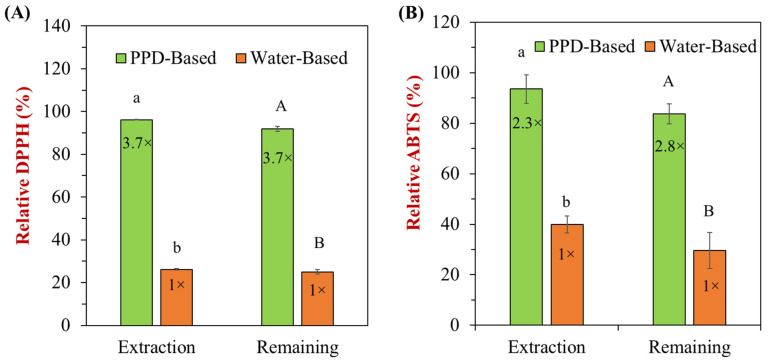
Impact of different treatments on the antioxidant properties of peanut skin-incorporated nanoemulsions: (**A**) relative DPPH (%) and (**B**) ABTS (%). Letters (a and b, or A and B) indicate significant differences between samples (*p* < 0.05). The ratio (e.g., “3.7×”) was calculated relative to the water-based sample.

The antioxidant properties of peanut skin-incorporated nanoemulsions were significantly enhanced using the PPD method compared to the water-based method, as demonstrated by two common antioxidant assays: DPPH (Figure 8A) and ABTS (Figure 8B). Both assays measured radical scavenging activity, which directly correlates with the antioxidant capacity of peanut skin-incorporated nanoemulsions. In the DPPH assay, the PPD-based nanoemulsions exhibited a substantial improvement, with approximately 3.7 times higher relative DPPH scavenging activity in both extraction and remaining antioxidant capacity compared to the water-based nanoemulsions. The PPD-based system maintained nearly 100% of the relative DPPH scavenging capacity, while the water-based system remained at about 27%. This indicates that the PPD method not only enhances antioxidant extraction from peanut skins but also preserves their activity during processing.

Similarly, in the ABTS assay, the PPD-based nanoemulsions showed 2.3 times higher relative ABTS activity during extraction and 2.8 times higher activity after processing compared to the water-based method. The PPD method efficiently extracted ABTS-related active compounds from peanut skins, achieving nearly 90% activity, while the water-based system showed significantly lower antioxidant capacity, with around 40% during extraction and 30% after overall processing. These findings strongly suggest that the PPD method is superior in both extracting and retaining the antioxidant properties of polyphenols from peanut skins when incorporated into nanoemulsions. The enhanced DPPH and ABTS activities underscore the potential of the PPD method to improve the functional quality of food systems by maximizing antioxidant capacity.

## 4. Conclusions

In this study, we advanced the conventional pH-driven method by optimizing operating conditions. Its simplicity and scalability, along with its ability to minimize degradation under controlled pH conditions, make it a promising approach for incorporating polyphenols into food systems. As a result, this method was used to encapsulate pH-sensitive quercetin into nanoemulsions with a high encapsulation efficiency (~95.9%). We also successfully demonstrated the effectiveness of the PPD method in upcycling polyphenols from byproducts, such as peanut skins, into nanoemulsions as a representative food system. The incorporation of polyphenols from peanut skin into the nanoemulsions resulted in a color change from white to pink and a slight increase in particle size. However, the highly negative zeta potential indicated that the PPD method preserved the colloidal stability of the nanoemulsions, ensuring long-term product quality. Importantly, the PPD method enhanced both the extraction and remaining efficiency of total polyphenolic content, for example, it achieved up to 1.8 times higher remaining efficiency compared to the conventional water-based method. Additionally, the PPD-based nanoemulsions exhibited improved antioxidant properties, with DPPH and ABTS activities increasing by 3.7 and 2.8 times, respectively, compared to the water-based approach. These findings highlight the PPD method as a versatile and efficient platform for developing polyphenol-enriched foods, improving their nutritional and functional value, while promoting more sustainable food processing practices by upcycling plant polyphenols from byproducts and increasing processing efficiency. However, further research is still essential to explore how polyphenols are solubilized within food systems, such as investigating whether they predominantly interact with biomolecules or remain in the oil phase. Moreover, additional experiments are needed to confirm whether it will affect the acceptability of polyphenol-enriched foods due to the potential bitterness of polyphenols and whether it has enhanced health benefits when applying the improved pH-driven method. It is also noted that we are unclear of the scalability for industrial applications. Future research will need to focus on how to effectively adapt the PPD method to industrial processes, in particular the key bottlenecks affecting its practical application on a larger scale.

## Data Availability

The original contributions presented in the study are included in the article, further inquiries can be directed to the corresponding author.
